# Establishment of combined diagnostic models of Alzheimer’s disease in a Chinese cohort: the Chongqing Ageing & Dementia Study (CADS)

**DOI:** 10.1038/s41398-022-02016-7

**Published:** 2022-06-16

**Authors:** Dong-Yu Fan, Jie-Ming Jian, Shan Huang, Wei-Wei Li, Ying-Ying Shen, Zhen Wang, Gui-Hua Zeng, Xu Yi, Wang-Sheng Jin, Yu-Hui Liu, Fan Zeng, Xian-Le Bu, Li-Yong Chen, Qing-Xiang Mao, Zhi-Qiang Xu, Jin-Tai Yu, Jun Wang, Yan-Jiang Wang

**Affiliations:** 1 Department of Neurology, Daping Hospital, Third Military Medical University, Chongqing, China; 2 Chongqing Key Laboratory of Ageing and Brain Diseases, Chongqing, China; 3 Shigatse Branch, Xinqiao Hospital, Third Military Medical University, Shigatse, China; 4 First Clinical Medical College, Shanxi Medical University, Taiyuan, China; 5 Department of Neurology, First Affiliated Hospital, Shanxi Medical University, Taiyuan, China; 6 Department of Neurology, Western Theater General Hospital, Chengdu, China; 7 Department of Critical Care Medicine, Daping Hospital, Third Military Medical University, Chongqing, China; 8 Department of Anaesthesiology, Daping Hospital, Third Military Medical University, Chongqing, China; 9 Department of Neurology and Institute of Neurology, Huashan Hospital, State Key Laboratory of Medical Neurobiology and MOE Frontiers Center for Brain Science, Shanghai Medical College, Fudan University, Shanghai, China; 10 State Key Laboratory of Trauma, Burn and Combined Injury, Institute of Surgery Research, Daping Hospital, Third Military Medical University, Chongqing, China; 11 Center for Excellence in Brain Science and Intelligence Technology, Chinese Academy of Sciences, Shanghai, China

**Keywords:** Diagnostic markers, Learning and memory

## Abstract

Cerebrospinal fluid (CSF) biomarkers are essential for the accurate diagnosis of Alzheimer’s disease (AD), yet their measurement levels vary widely across centers and regions, leaving no uniform cutoff values to date. Diagnostic cutoff values of CSF biomarkers for AD are lacking for the Chinese population. As a member of the Alzheimer’s Association Quality Control program for CSF biomarkers, we aimed to establish diagnostic models based on CSF biomarkers and risk factors for AD in a Chinese cohort. A total of 64 AD dementia patients and 105 age- and sex-matched cognitively normal (CN) controls from the Chongqing Ageing & Dementia Study cohort were included. CSF Aβ42, P-tau181, and T-tau levels were measured by ELISA. Combined biomarker models and integrative models with demographic characteristics were established by logistic regression. The cutoff values to distinguish AD from CN were 933 pg/mL for Aβ42, 48.7 pg/mL for P-tau181 and 313 pg/mL for T-tau. The AN model, including Aβ42 and T-tau, had a higher diagnostic accuracy of 89.9%. Integrating age and *APOE* ε4 status to AN model (the ANA’E model) increased the diagnostic accuracy to 90.5% and improved the model performance. This study established cutoff values of CSF biomarkers and optimal combined models for AD diagnosis in a Chinese cohort.

## Introduction

Alzheimer’s disease (AD) is the most common type of dementia in the elderly, which is characterized by amyloid plaque comprised of amyloid-β (Aβ) and neurofibrillary tangles comprised of hyperphosphorylated tau [1–3]. Currently, disease-modifying therapies for AD are still lacking [4], and clinical trials of drugs targeting the pathological aspects have suffered serious setbacks, partially due to late intervention time and inaccurate clinical diagnosis [5]. Our group and others previously reported that 9–35% of patients clinically diagnosed with probable AD were Aβ negative [6–9], whereas 26–33% of cognitively normal elderly were Aβ positive in the brain [10–12]. Therefore, accurate AD diagnosis is critical for successful therapy development.

Biomarkers are essential to establish an accurate diagnosis and provide objective evidence for monitoring disease progression and evaluating drug efficacy. In recent years, the “ATN (Amyloid/Tau/Neurodegeneration)” framework of AD biomarkers has been proposed and integrated into AD diagnostic criteria by the NIA-AA [13]. Molecular imaging techniques (e.g., Aβ-PET) provide in vivo pathological evidence for AD patients [14]. However, their clinical applications are limited due to high costs and limited accessibility. CSF biomarkers are relatively cost effective and more accessible. Studies in western populations have shown the good diagnostic performance of CSF biomarkers, including Aβ42, phosphorylated tau 181 (P-tau181), and total tau (T-tau), with 85–90% specificity and sensitivity in patients with Alzheimer’s dementia [15, 16].

However, substantial variability in measured biomarker levels was found due to differences in pre-analysis procedures, assay methods, as well as ethnicity [17, 18]. Currently, there are no uniform cutoff values of these markers for diagnostic criteria in Chinese population. As a member of the Alzheimer’s Association Quality Control (QC) program for CSF biomarkers [19], we established diagnostic cutoff values of CSF core biomarkers for AD in a Chinese cohort using the methods recommended by the QC program, and proposed an optimal diagnostic model of combined CSF biomarkers. This study is a step toward identifying uniform cutoff values for the Chinese population to enable the introduction of CSF biomarkers into clinical practice.

## Materials and methods

### Participants

AD patients and age- and sex-matched controls with normal cognition in this study were enrolled from the Chongqing Ageing & Dementia Study (CADS) cohort. All participants were recruited from Chongqing Daping Hospital between January 2015 to March 2021. Individuals were excluded for the following reasons: (1) concomitant neurologic disorder (multiple sclerosis, Parkinson’s disease, epilepsy, metabolic encephalopathy, hydrocephalus, etc.); (2) severe systemic diseases (liver insufficiency, renal insufficiency, cancer, special infections, etc.); (3) enduring mental illness (e.g., schizophrenia); (4) refusal of lumbar puncture and blood sampling; (5) unable to comply with the cognitive examination. This study was approved by the Institutional Review Board of Daping Hospital, and all participants and their caregivers provided informed consent.

### Clinical assessments and diagnosis of AD dementia

The clinical assessments and diagnosis of AD dementia were performed following our previous protocol [20]. In short, the demographic characteristics (including age, sex, education level), history of present illness, medical history (including diabetes, hypertension, dyslipidemia, coronary heart disease, etc.) and medication use were collected. Then, all participants underwent clinical assessments including physical examination, laboratory tests, *APOE* genotyping, magnetic resonance imaging, and neuropsychological tests. Diagnosis of AD dementia was made according to the criteria of the National Institute of Neurological and Communicative Disorders and Stroke and the Alzheimer’s Disease and Related Disorder Association (NINCDS-ADRDA) [21].

### CSF sampling and processing

CSF samples were collected by lumbar puncture and processed according to a standard procedure [22]. Specifically, the CSF samples without visible blood contamination were collected in polypropylene tubes, followed by centrifugation at 2000 × *g* for 10 min at room temperature within 2 h. The supernatant was aliquoted and stored frozen at −80 °C until analysis.

### Measurements of CSF biomarkers

CSF Αβ42 levels were determined using sandwich ELISA (INNOTEST^®^ β-AMYLOID (1–42), Fujirebio, Belgium). CSF levels of total tau and P-tau181 were determined using sandwich ELISA INNOTEST hTau Ag, and INNOTEST PHOSPHO-TAU (181), respectively. All measurements were performed by an experienced laboratory technician who was blinded to the clinical information.

### Statistical analysis

Sample size calculations were performed in PASS 11.0 software (NCSS, Kaysville, USA). In accordance with the estimation method of sample content for diagnostic test evaluation, we defined that Power (1-beta) = 0.95, alpha = 0.05, R (ratio of control to case group sample cases) = 2:1, AUC0(AUC to be achieved) = 0.7, AUC1(AUC from previous information) = 0.85, Type of data = continuous, Alternative Hypothesis = two-sided test. The results showed 52 cases in the case group and 104 cases in the control group.

The data are expressed as mean ± standard deviation (SD) or median (interquartile range, IQR) for numerical variables or as the count (%) for categorical variables. The differences in demographic characteristics and CSF biomarker levels between AD and control groups were assessed with two-tailed independent *t*-test, Mann Whitney U test, or *χ*^2^ test as appropriate. Spearman correlation analyses were used to examine the correlations between mini-mental state examination (MMSE) scores and CSF biomarkers levels.

The receiver operating characteristic curve (ROC) analysis is used to evaluate the diagnostic value of CSF biomarkers. The area under the curve (AUC), Akaike information criterion (AIC), sensitivity, specificity, accuracy, and the diagnostic cutoffs were estimated according to the largest Youden index. The combined diagnostic model of CSF biomarkers (we named it CSF index) was established by logistic regression (enter method), with Aβ42 (A), P-tau181 (T), and T-tau (N) as independent variables. Moreover, demographic information, including age (A’), sex (S), and *APOE* ε4 status (E), is added incrementally to the optimal model by logistic regression (forward method). Specifically, one demographic indicator is added at each step on the principle of the lowest AIC, and the process is repeated at the next step until the AIC does not decrease any further. AUC, AIC, and accuracy were calculated for each model. The DeLong test was used to compare the statistical significance between ROC curves [23]. Internal validation was performed by 1000 bootstrapped trials to evaluate the fitted degree among our apparent model, the Bias-corrected model, and the ideal model; the mean absolute error (MAE) < 0.05 meant high fitted degree.

All hypothesis testing was two-sided, *p* < 0.05 was defined as statistically significant. The computations were performed using SPSS 26.0 software (IBM SPSS Inc., Chicago, USA) and the R programming language (version 4.1.1).

## Results

### Characteristics of the study population

A total of 64 AD dementia patients and 105 age- and sex-matched cognitively normal (CN) controls were included in this study. The characteristics of these participants are shown in Table 1. There were no significant differences in age and sex between the two groups. The proportion of *APOE* ε4 carriers was higher in the AD group (*p* = 0.001). AD group had lower education levels and MMSE scores (*p* < 0.001).

**Table 1:** Characteristics of the participants.

Characteristics	Controls (*n* = 105)	AD (*n* = 64)	*p-*value
Age, mean (SD), y	65.02(9.03)	65.53(8.91)	0.720
Male, *n* (%)	57(54.29)	31(48.44)	0.460
*APOE* ε4 carriers, *n* (%)	20(19.05)	29(45.31)	**0.001**
Education level, median (IQR), y	12(9–12)	9(6–9)	**<0.001**
MMSE score, median (IQR)	28(26–29)	14(11–17)	**<0.001**
Comorbidities, *n* (%)			
Diabetes	9(8.57)	12(18.75)	0.052
Hypertension	29(27.62)	20(31.25)	0.614
Dyslipidemia	3(2.86)	17(26.56)	**<0.001**
Coronary artery disease	7(6.67)	12(18.75)	**0.016**
CSF Aβ42, median (IQR), pg/mL	1385.97(1136.24–1653.33)	607.79(444.82–753.22)	**<0.001**
CSF T-tau, median (IQR), pg/mL	175.51(135.71–261.68)	405.89(243.21–615.84)	**<0.001**
CSF P-tau181, median (IQR), pg/mL	43.01(35.42–52.83)	59.38(43.98–104.73)	**<0.001**

Two-tailed independent *t*-tests or Mann–Whitney U test as appropriate.

*APOE* *ε4* apolipoprotein E ε4 allele, *MMSE* mini-mental state examination*, CDR* clinical dementia rating, *CSF* cerebrospinal fluid, *IQR* interquartile range.

Bold values indicate statistically significant *p* values less than 0.05.

### Cutoffs of core CSF biomarkers

**Fig. 1:**
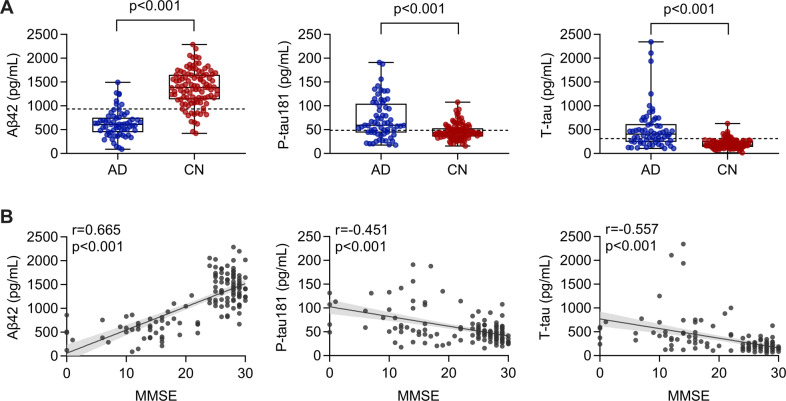
Comparison of single CSF biomarkers and their correlation with MMSE scores. **A** Comparison of CSF Aβ42, P-tau181, and T-tau between AD (*n* = 64) and CN (*n* = 105) group. Boxes represent the 25th, 50th, and 75th data percentiles. Whiskers represent the lowest and highest data. The dashed lines indicate the cutoff values for each biomarker. **B** Correlations between CSF Aβ42, P-tau181, T-tau, and MMSE scores. The best-fit linear regression line is shown and 95% confidence intervals are superimposed. MMSE, mini-mental state examination; AD, Alzheimer’s disease; CN, cognitively normal.

Compared with controls, AD dementia patients had significantly lower Aβ42 levels, higher P-tau181 and T-tau levels in CSF (*p* < 0.001) (Fig. 1A, Table 1). The differences remained significant after adjusting for *APOE* ε4 status, education level, and comorbidities (*p* < 0.05). MMSE scores were positively associated with CSF Aβ42 (*r* = 0.665, *p* < 0.001), and negatively with P-tau181 (*r* = −0.451, *p* < 0.001) and T-tau (*r* = −0.557, *p* < 0.001) (Fig. 1B).

ROC analyses were performed to determine the diagnostic values of single CSF biomarkers. The cutoff value of CSF Aβ42 to distinguish AD from CN was 933 pg/mL (A^+^: Aβ42 < 933 pg/mL), with AUC of 94.0% (95%CI: 90.4–97.5%), the sensitivity of 89.1% and specificity of 87.6%. The diagnostic accuracy of Aβ42 was 88.2%. The cutoff values of P-tau181 and T-tau were 48.7 pg/mL and 313 pg/mL (T^+^: P-tau181 > 48.7 pg/mL; N^+^: T-tau>313 pg/mL), with AUC of 70.3% and 83.2%, respectively. The diagnostic accuracies were 69.2% for P-tau181 and 81.1% for T-tau, lower than that of Aβ42 (Table 2).

**Table 2:** Performance of CSF biomarker cutoffs.

CSF biomarkers	Cutoff	AUC (95% CI), %	AIC	Sensitivity, %	Specificity, %	Accuracy, %
Aβ42	<933 pg/mL	94.0 (90.4, 97.5)	106.41	89.1	87.6	88.2
P-tau181	>48.7 pg/mL	70.3 (61.0, 79.1)	197.40	70.3	68.6	69.2
T-tau	>313 pg/mL	83.2 (75.3, 89.3)	160.50	65.6	90.5	81.1
AT	>−0.461	94.3 (90.9, 97.6)	104.97	85.9	90.5	88.8
AN	>−0.368	94.9 (91.9, 98.0)	99.22	90.6	89.5	89.9
TN	>36.3	82.5 (75.6, 89.4)	156.40	68.8	87.6	80.5
ATN	>−0.173	95.0 (92.0, 98.1)	100.91	89.1	91.4	90.5
ANA’	>0.332	95.5 (92.5, 98.6)	95.47	87.5	92.4	90.5
ANA’E	>−0.401	96.0 (93.2, 98.9)	93.31	90.6	90.5	90.5
ANA’ES	>−0.421	96.2 (93.2, 99.1)	94.67	92.2	89.5	90.5

*CSF* cerebrospinal fluid, *AUC* area under the curve, *AIC* Akaike information criterion, *CI* confidence intervals.

A, Aβ42; T, P-tau181; N, T-tau; A’, age; E, apolipoprotein E ε4 allele (*APOE* ε4) status.

AT = 3.944–0.006A + 0.018T; AN = 3.164–0.005A + 0.004N; TN = −2.629–0.034T + 0.14N; ATN = 3.392–0.005A–0.009T + 0.005N; ANA’ = 8.003–0.005A + 0.006N–0.075A’; ANA’E = 7.803–0.005A + 0.006N–0.079A’ + 1.008E; ANA’ES = 7.66–0.005A + 0.006T–0.081A + 1.024E + 0.459S (established by logistic regression).

**Fig. 2:**
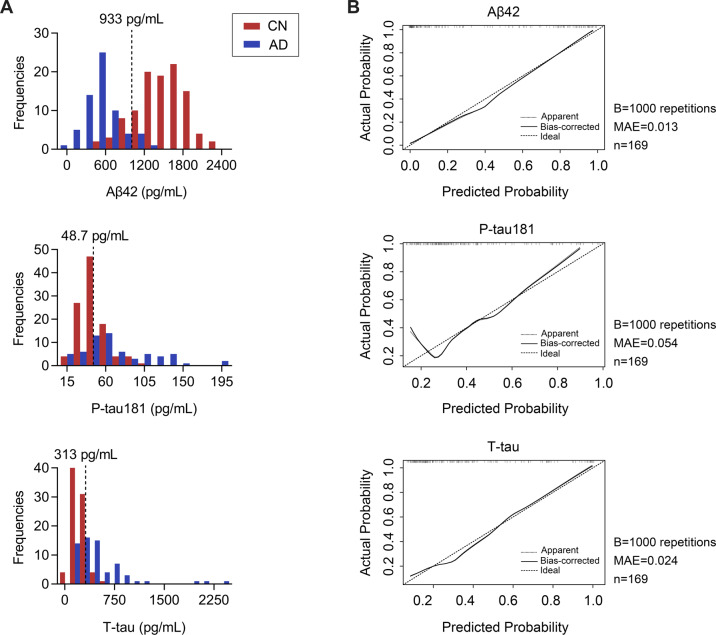
Frequency distribution and internal validation of single CSF biomarkers. **A** Frequency distribution of CSF Aβ42, P-tau181, and T-tau. The dashed vertical lines indicate the cutoff value for each biomarker. **B** The bootstrap-validated of CSF Aβ42, P-tau181, and T-tau. The Y-axis indicates the actual probability of AD and the X-axis indicates the predicted probability of AD. The 45-degree black dotted line represents the ideal prediction; the solid black line surrounding the 45-degree black dotted line represents the bias-corrected prediction; the black dotted line surrounding the 45-degree black dotted line represents the apparent prediction. AD, Alzheimer’s disease; CN, cognitively normal; MAE, mean absolute error.

To further verify whether the cutoffs of CSF biomarkers can distinguish AD dementia patients from CN intuitively, we further analyzed their frequency distribution. As shown in Fig. 2A, the distribution of Aβ42 levels was in good agreement with the classification of the disease status, showing a bimodal distribution.

Internal validation was performed using bootstrapping with 1000 repetitions to evaluate the reliability of CSF biomarkers. The results showed that Aβ42 and T-tau had a high fitted degree among our apparent model, the Bias-corrected model, and the ideal model (Aβ42: MAE = 0.013; T-tau: MAE = 0.024), while P-tau181 had a medium fit (MAE = 0.054) (Fig. 2B), indicating the reliability of the diagnostic efficacy of Aβ42 and T-tau.

### Combined models of CSF biomarkers

**Fig. 3:**
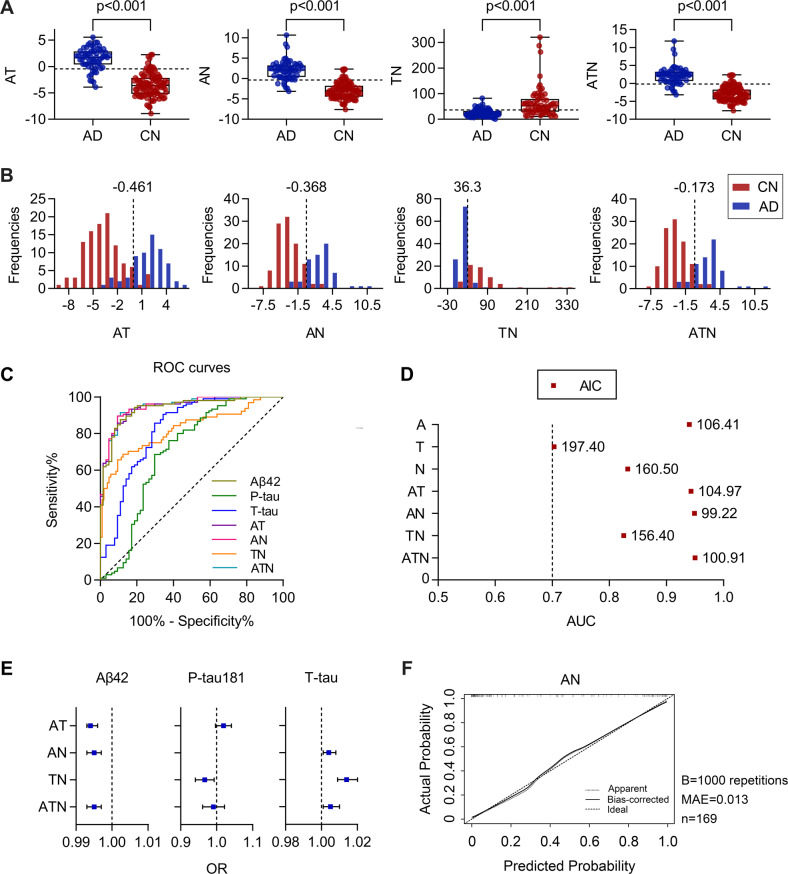
Combined models of CSF biomarkers. **A** Comparisons of AT, AN, TN, and ATN model indices between AD (*n* = 64) and CN (*n* = 105) group. Boxes represent the 25th, 50th, and 75th data percentiles. Whiskers represent the lowest and highest data. The dashed lines indicate the cutoff value for each index. **B** Frequency distribution of AT, AN, TN, and ATN model indices. The dashed vertical lines indicate the cutoff values for each index. **C** ROC curves of CSF biomarkers and combined model indices. **D** AUC (x-axis) and AIC values (numbers in plots) for each biomarker and combined biomarker model. The dashed vertical line shows AUC = 0.7. **E** The OR values represent the contribution of each biomarker to the combined models. The error bars represent 95% confidence intervals. **F** The bootstrap-validated of CSF AN index. The Y-axis indicates the actual probability of AD and the X-axis indicates the predicted probability of AD. A, Aβ42; T, P-tau181; N, T-tau; AD, Alzheimer’s disease; CN, cognitively normal; ROC, receiver operating characteristic curve; AUC, area under the curve; AIC, Akaike information criterion; OR, odds ratio; MAE, mean absolute error.

To improve the accuracy of AD diagnosis, we established combined diagnostic models of CSF biomarkers, including AT, AN, TN, and ATN, by logistic regression (see Table 2 for details). Compared with the controls, AD group had significantly higher AT, AN, ATN indices, and lower TN index (*p* < 0.001) (Fig. 3A), even after adjusting for *APOE* ε4 status, education level, and comorbidities (*p* < 0.05). The frequency distributions of AT, AN, and ATN showed a good agreement with the classification of the disease status (Fig. 3B).

ROC analyses were performed to determine the AD diagnostic accuracy of each model; the lowest AIC, the best tradeoff between model fit and model complexity, was used to select the optimal model. As shown in Table 2 and Fig. 3, among the single biomarkers and combined biomarker models, CSF Aβ42 alone and the AN and the ATN models had higher and similar AUCs by DeLong test (*p* > 0.05); whereas the AN model had the lowest AIC, indicating the best diagnostic performance. The cutoff value of AN index to distinguish AD from control was −0.368, with an AUC of 94.9% (95%CI: 91.9–98.0%), a sensitivity of 90.6% and a specificity of 89.5%. The internal validation indicated that the AN model was reliable for AD diagnosis (MAE = 0.013) (Fig. 3F).

### Integrative models of demographic characteristics with CSF biomarkers

**Fig. 4:**
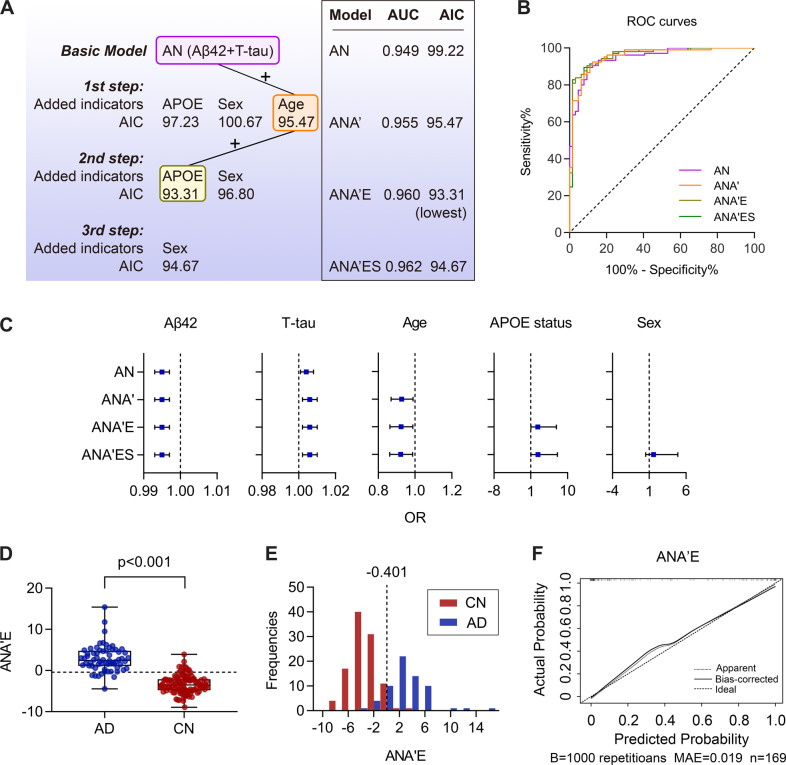
Integrative models of demographic characteristics with CSF biomarkers. **A** Model selection process. The data-driven model was selected with the lowest AIC (ΔAIC > 2). Based on the AN model, age, sex, and *APOE* ε4 status were added in a stepwise procedure, the model with lower AIC was obtained by adding one indicator at each step. **B** ROC Curves of integrated model indices. **C** OR values represent the contribution of each indicator to the integrated models. The error bars represent 95% confidence intervals. **D** Comparison of ANA’E index between AD (*n* = 64) and CN (*n* = 105) group. Boxes represent the 25th, 50th, and 75th data percentiles. Whiskers represent the lowest and highest data. The dashed lines indicate the cutoff value for ANA’E index. **E** Frequency distribution of ANA’E index. The dashed vertical lines indicate the cutoff values for ANA’E index. **F** The bootstrap-validated of CSF ANA’E index. The Y-axis indicates the actual probability of AD and the X-axis indicates the predicted probability of AD. A, Aβ42; T, P-tau181; N, T-tau; A’, age; E, *APOE* ε4 status; S, sex; *APOE* ε4, apolipoprotein E ε4 allele; ROC, receiver operating characteristic curve; AUC, area under the curve; AIC, Akaike information criterion; OR, odds ratio; AD, Alzheimer’s disease; CN, cognitively normal; MAE, mean absolute error.

Age, sex, and *APOE* genotype are associated with the risk of AD, so we investigated whether integrating demographic information could improve the diagnostic efficacy of CSF biomarker models. A data-driven model selection was performed to select the optimal model with the lowest AIC. The AN model, the best-combined biomarker model, was used as the basis; then age, sex, and *APOE* ε4 status were added in a stepwise procedure to examine the performance of integrative models. Better model performance was defined as being at least two AIC points lower than the previous model (ΔAIC > 2) [24] (Fig. 4A). The addition of demographic information slightly increased the AUCs and accuracy although no significant differences were detected by the DeLong test (*p* > 0.05) (Fig. 4B). The first step generated the ANA’ model (A’: age) with the AIC of 95.47 and AUC of 95.5%; The second step generated the ANA’E (E: *APOE* ε4 status) model, with AIC of 93.31 and AUC of 96.0% (95%CI: 93.2–98.9%). In the third step added sex, the AIC no longer decreased in ANA’ES (S: sex) model, with the higher AIC of 94.67 and AUC of 96.2%. Therefore, ANA’E had the lowest AIC in the above models, indicating the best diagnostic performance. The cutoff value of ANA’E model index was −0.401, able to well distinguish the two populations. The diagnostic accuracy of ANA’E model was up to 90.5% (Fig. 4D, E). The internal validation also confirmed the reliability of the ANA’E model (MAE = 0.019) (Fig. 4F).

## Discussion

In this study, we defined the cutoff values of CSF Aβ42, P-tau181, and T-tau for AD diagnosis (A+: Aβ42 < 933 pg/mL; T+: P-tau181 > 48.7 pg/mL; N+: T-tau > 313 pg/mL) in a Chinese cohort. Among these single biomarkers, CSF Aβ42 had the highest diagnostic accuracy of 88.2% in distinguishing AD patients from cognitively normal participants. Among the combined models of CSF biomarkers, AN was the simplest model while showing good diagnostic performance, with an accuracy of 89.9% (cutoff value > −0.368). In addition, it makes sense to integrate age and *APOE* ε4 status in the model to increase the accuracy (90.5%) and performance of the diagnosis.

The diagnosis of AD has now moved into the pathological phase with the inclusion of CSF biomarkers and amyloid PET in international guidelines [25, 26]. Although amyloid PET is the intuitive marker of amyloid pathology, it reflects the accumulation of sufficient amyloid to form an amyloid PET signal over many years. Whereas CSF biomarkers show the state of production and clearance of Aβ42 [27], and are likely to be positive early in the course of the disease before sufficient amyloid has accumulated, making it important in early diagnosis of AD [28–30]. Studies in recent years have suggested that blood-based biomarkers (e.g., P-tau181, P-tau217, P-tau231, etc.) enable the diagnosis and prediction of AD [31–33]. However, some studies disagree with this [34]. Therefore, even with great advances in amyloid PET and blood biomarkers, research on CSF biomarkers is still necessary.

Over the last decade, different assay methods have been developed for CSF biomarkers [35]. However, there are no international standardized cutoffs yet [36]. For the INNOTEST ELISA method used in our present study, the cutoff of Aβ42 was previously reported to be 368–875 pg/mL, cutoffs of T-tau and P-tau181 were 289–353 pg/mL and 54–65 pg/mL, respectively [37–42]. The AUC for Aβ42 in these studies ranged from 85 to 93%. The variation between laboratories is mainly due to pre-analytical and analytical factors, as well as racial differences. In this study, pre-analytical factors, including fasting, tube types, centrifugation, storage temperature and time, were strictly followed the guidelines from Alzheimer’s Biomarkers Standardization Initiative (ABSI) and the Alzheimer’s Association [43, 44], and consistent with the Standard Operating Procedure (SOP) [45]. For the analysis process, our laboratory is one of the centers of the Alzheimer’s Association external quality control (QC) program (code Lab129) [19, 46]. The majority of the laboratories in the program use INNOTEST ELISA test kits [47]. Among them, T-tau and P-tau181 levels measured in our lab are in the middle, while Aβ42 level is the third-highest, but still within the quality control range. The results of repeated tests are stable in our lab, suggesting that the measurements of CSF biomarkers in our laboratory are reliable.

Previous studies have found that combining tau with Aβ42 can improve diagnostic accuracy, such as the tau/Aβ42 ratio. In this study, we used a more precise approach to obtain a combined model by logistic regression and found that AN was the best model. Compared to Aβ42 alone, the AN model improves the sensitivity and specificity and reduces false positives and false negatives. The AUC of the AN model was slightly higher than that of the AT model (94.9 vs. 94.3), probably because of the weak diagnostic performance of P-tau181 itself in this study. This may result from the fact that the onset of tau pathology precedes neurological damage, and the AD patients selected for this study were symptomatic with lower cognitive scores and were already in the later stages of the disease, when the “N” changes were more pronounced. Also, because there are natural fluctuations or variations in the production, secretion, and degradation of CSF proteins [48], the combined model reduces random errors or variance in measurements and compensates for the natural variations in the concentration. In addition, the *APOE* ε4 allele is the most powerful genetic risk factor for sporadic AD and has been shown to influence CSF Aβ and tau levels; as well, ageing and female are also major risk factors of AD [49–52]. Hence previous studies have suggested different diagnostic cutoffs for different age groups and *APOE* ε4 status [53–55]. Recent studies on blood biomarkers have suggested that models incorporating age, sex, and *APOE* genotype could improve the diagnostic prediction of AD [56, 57]. In our study, the addition of age and *APOE* ε4 status to the combined biomarker model (ANA’E) could increase the AUC from 95.0% to 96.0%, and significantly improve the model’s performance. Therefore, when the patient’s age and *APOE* ε4 status are available, the ANA’E model would be a better choice.

Recent perspectives propose that the addition of an “X” to the ATN framework could reflect the whole spectrum of AD pathologies [58]. The “X” represents biomarkers associated with synaptic damage, apoptosis, oxidative stress, neuroinflammation, neuroimmunity, mitochondrial dysfunction, and unrealized pathologies of AD [59]. An integrated model based on the ATXN framework could be applied not only for diagnosis, differential diagnosis, and prognosis, but also for the treatment and related trials of AD. Since the network of pathophysiology is complex and full of interconnections, all the dimensions in the framework should be involved in cocktail therapy. However, there are some challenges before widespread use of the ATXN framework. Large multicenter studies are still required to validate and standardize these biomarkers and their cutoffs, and the accuracy of biomarkers in the ATXN framework needs to be improved based on ultrasensitive technologies. Clarification of the interacting mechanisms of these biomarkers furthermore can provide the theoretical foundation for the application of the ATXN framework.

There are some limitations to this study. First, due to the difficulty of collecting CSF from AD dementia patients, the sample size of this study was relatively small. Even though internal validation has been performed, there’s still a need to expand the sample size in the external validations. Second, the participants enrolled were clinically diagnosed and lacked pathological evidence of Aβ-PET. Adequate validation in sufficient Aβ+ AD patients and Aβ− controls is highly needed before the findings of this study can move toward clinical practice, which requires further expansion of the sample size and inclusion of more stringent diagnostic criteria based on Aβ-PET in the follow-up. Finally, the assessment and external validation of the differential diagnostic ability is equally important before entering clinical practice, and we need to include more patients with other types of dementia to validate the differential diagnostic efficacy of the model in the future.

In conclusion, this study established the cutoff values of CSF biomarkers for AD diagnosis in a Chinese cohort, which is essential for the clinical application of AD biomarkers in Chinese population.
